# Mapping Lymphatic Drainage from the Human Brain to the Head and Neck: Potential Surgical Implications for Dementia

**DOI:** 10.1055/a-2897-0477

**Published:** 2026-08-21

**Authors:** Dung H. Nguyen, Carmen Higueras, Hiroo Suami, Anna Carrera Burgaya, Joon Pio Hong

**Affiliations:** 1 Division of Plastic and Reconstructive Surgery 6429 Stanford UniversityStanfordCaliforniaUnited States; 2 Department of Plastic Surgery 16514 Hospital Universitari Germans Trias i PujolBadalonaSpain; 3 School of Health Sciences and Nursing, Faculty of Medicine, Health and Human SciencesAustralian Lymphoedema Education, Research and Treatment Centre (ALERT), Macquarie UniversitySydneyNew South WalesAustralia; 4 Clinical Anatomy, Embryology and Neuroscience Research Group (NEOMA), Department of Medical SciencesUniversity of Girona School of MedicineGironaSpain; 5 Department of Plastic Surgery 35029 Asan Medical Center, University of UlsanSeoulSeoulKorea (the Republic of)

**Keywords:** others, pediatric/craniomaxillofacial/head and neck, reconstruction/lymphedema, lymphedema-lymphatic physiology, cervical lymph nodes, meningeal lymphatic system, Alzheimer's disease, lymphatic reconstruction, lymph node-to-vein anastomosis

## Abstract

**Background:**

Lymphatic surgery of the head and neck has been proposed as a potential approach to prevent, delay, or treat Alzheimer's disease (AD). This exploratory anatomical study undertook to map and evaluate potential targets for surgical feasibility studies.

**Methods:**

This exploratory anatomical and preliminary laboratory study was supported by fresh cadaver dissection, with indocyanine green (ICG) lymphography to visualize the drainage pathway. Immunohistochemistry was used to confirm the presence of lymphatic tissues and to detect β-amyloid in lymphatic tissues from a cadaver with confirmed dementia. A simulated lymph node-to-vein anastomosis (LNVA) was performed using robotic-assisted supermicrosurgery with the Symani Surgical System (Medical Microinstruments, Inc., Jacksonville, FL, United States).

**Results:**

After injection of ICG at the dural meninges, fluorescence progressed toward the skull base to the Level II deep cervical lymph nodes (dCLNs). When injected at the parietal lymphatics, ICG drained past the deep digastric point to the Level II cervical lymph nodes (CLNs). Veins localized adjacent to dCLNs at the deep digastric point and Level II were suggested as candidate surgical targets for LNVA. Histology of lymph nodes from a dementia-confirmed cadaver identified β-amyloid at Level II dCLNs but not at lower levels or in the groin. A robotic-assisted microsurgical LNVA was shown to be feasible.

**Conclusions:**

This study found evidence supporting a posterior pathway from human dural meninges to the dCLNs and identified potential surgical candidates for AD intervention. Further research is warranted to build on the feasibility of this approach.

## Introduction

The functional and anatomical links between the mammalian brain's waste-clearance mechanisms, the network of lymphatic vessels infiltrating the dural layer of the meninges, and the extracranial lymphatic system are currently a topic of considerable interest, especially given their implication in neurological disorders and neurodegenerative diseases. 1 2 3 It has previously been hypothesized, based on multiple lines of evidence, that partial or total occlusion of the proposed lymphatic drainage route for waste-laden cerebrospinal fluid (CSF) could impede removal of neurotoxic macromolecules from the brain and play a role in the pathogenesis of Alzheimer's disease (AD) and other disorders of cognition and brain function. 1 2 3 4 5 6 7 8 9 10 11 12 13 14 15 16 Together with the hypothesis that lymphedema is a pervasive systemic disorder, with effects distributed throughout the body, 17 this has led our group and others to propose that lymphatic surgery to bypass the obstruction could be feasible as a surgical approach for prevention, delay, or potential treatment of AD. 2 18 19 20 21

The relationship between the central nervous system (CNS) and the extracranial lymphatic system is intriguing. However, much of the current anatomical and physiologic understanding of the drainage pathways is dominated by evidence from rodent models. 1 3 22 Study of the pathway in humans, primarily through imaging and biomarker studies, has shown that there are potentially important differences between rodent and human cerebral drainage pathways. 1 22 23 24 25 26 For example, in both rodents and humans, the CSF outflow from the brain is known to cross the cribriform plate and drain through the nasal lymphatics (the anterior pathway) and to drain posteriorly through the jugular foramen to the deep cervical lymph nodes (dCLNs) (the posterior pathway). However, it has also been shown that the spinal meningeal pathway drains to the intercostal or paravertebral lymph nodes in humans but not in mice. 27 This difference may result in a different balance of CSF outflow between species—the magnitude and importance of which have not yet been determined.

This and other differences between mouse and human drainage pathways are a reminder that any surgical solution for lymphatic obstruction of the head and neck must be developed based on direct empirical evidence from humans. There are many open questions. Besides the relative importance of the human anterior and posterior drainage pathways, we have yet to understand the anatomy of the posterior route between the skull base and the digastric muscle, 28 and the spatial relationships between the cervical lymphatics and the small veins of the head and neck. The answers are critical to the future development of surgical targets (e.g., the relevant lymphatic vessels, adjacent veins, and specific lymph nodes) that can be used to create anatomically appropriate pathways for increased cerebral CSF drainage after lymphovenous anastomosis (LVA) or lymph node-to-vein anastomosis (LNVA).

Confirmation of the specific human drainage pathways is also essential to understand potential safety issues and avoid causing unintended patient harm if physiological routes are bypassed. One concern is that the dCLNs function as tumor-draining lymph nodes for cancers of the head and neck. 29 Circumventing these critical immunological filters in an effort to halt progression of dementia could exchange that problem for a higher risk of future malignant metastasis. Another emergent safety concern is the potential association between indiscriminate surgical dissection of cervical lymph nodes (CLNs) and *higher* levels of dementia. 6 16 Thus, it is our opinion that a nuanced understanding of the continuity between dural and extracranial lymphatics will be essential to minimizing harm while maximizing outcomes.

The present report describes the results of an exploratory collaboration among various groups, aimed at exploring the anatomy and techniques needed to confirm the hypothesis of posterior drainage and supported by fresh cadaver dissection. The goal was to visualize and confirm the presence of dural lymphatics in humans, map the posterior drainage route in humans from the skull base to the dCLNs, and to evaluate potential surgical targets for LVA and LNVA feasibility studies.

## Methods

### Study Design and Ethics

This was an exploratory anatomical and preliminary laboratory study. Because of the preliminary nature of the experiments, the investigators had the freedom to perform techniques according to their preferred standard practices. Every effort was made to follow all local and international ethical guidelines and laws related to the use of human cadaveric donors in anatomical research. 30 31 Anatomical donors 32 were procured from Stanford University Center for Clinical Sciences Research; the Surgical Skills and Anatomy Centre, Faculty of Medicine, Health and Human Sciences at Macquarie University; and the Body Donation Program of the University of Girona. Legally required ethical oversight and donor privacy protections were provided by the appropriate governing body at each institution.

### Exposure and Visualization of Dural Lymphatics

To visualize the structures of the healthy human dural lymphatics, fresh-frozen (i.e., non-embalmed) cadavers ( *n* = 3) with no evidence of dementia were thawed and prepared for dissection. The scalp was incised with a sagittal midline incision and separated from the skull bone, together with the periosteum. The temporal muscle was attached to the scalp flap, and dissection was performed to the level of the zygomatic arch. After the cranium was exposed, the calvarium and dural membrane were cut horizontally with the bone saw and scalpel and then detached. The brain was removed, and the meninges of the skull base were exposed and prepared for the dye injection.

### Visualization of Lymphatic Vessels Using Hydrogen Peroxide

**Video S1** Method for the localization of lymphatic vessels through the injection of hydrogen peroxide into a lymph node.


Lymphatics were identified for cannulation either by direct visualization through the microscope or by locating the lymph nodes and injecting or dousing them topically with hydrogen peroxide, which causes them to swell as previously described ( Video S1 [available in the online version only]). 33 34 35 36 Please note that hydrogen peroxide injection should be performed only in a research setting, as it can chemically deactivate indocyanine green (ICG), resulting in reduced or loss of its characteristic absorbance and fluorescence properties. Accordingly, imaging must be conducted promptly after injection to minimize ICG degradation and preserve sufficient signal intensity for reliable detection and optimal visualization.

### Indocyanine Green Lymphography

The target sites for injection of ICG were a few centimeters separate from the branches of the meningeal arteries, as described previously. 37 A 34G needle and 1-mL Luer lock syringe were used for the injection. The tip of the needle was placed manually at the target site within the dura, and 0.1 mL of ICG (Diagnostic Green Ltd., Westmeath, Ireland) was gently injected. Successful injection was indicated by immediate visualization of a mesh-like venolymphatic structure using infrared fluorescent imaging (PDE Neo II, Hamamatsu Photonics, K.K., Hamamatsu, Japan).

### Mapping the Extracranial Lymphatics of the Posterior Cerebral Outflow Pathway

A separate set of fresh cadaveric heads ( *n* = 3), also from healthy donors, was used for experiments mapping the lymphatic pathway from the jugular foramen to the cervical lymphatics through the posterior route and for identifying the involved lymph nodes at the different cervical levels. The arteries and veins were prestained to help distinguish them from lymphatic vessels. The platysma was resected, and the sternocleidomastoid muscle (SCM) was retracted to expose the internal jugular vein and lymph node chains from the digastric muscle to the clavicle ( Fig. 1 ). This area of the neck corresponds to the lymph node Levels II (jugulodigastric dCLN), III and VA (superior jugular dCLN), as well as IV and VB (inferior jugular dCLN).

**Fig. 1:**
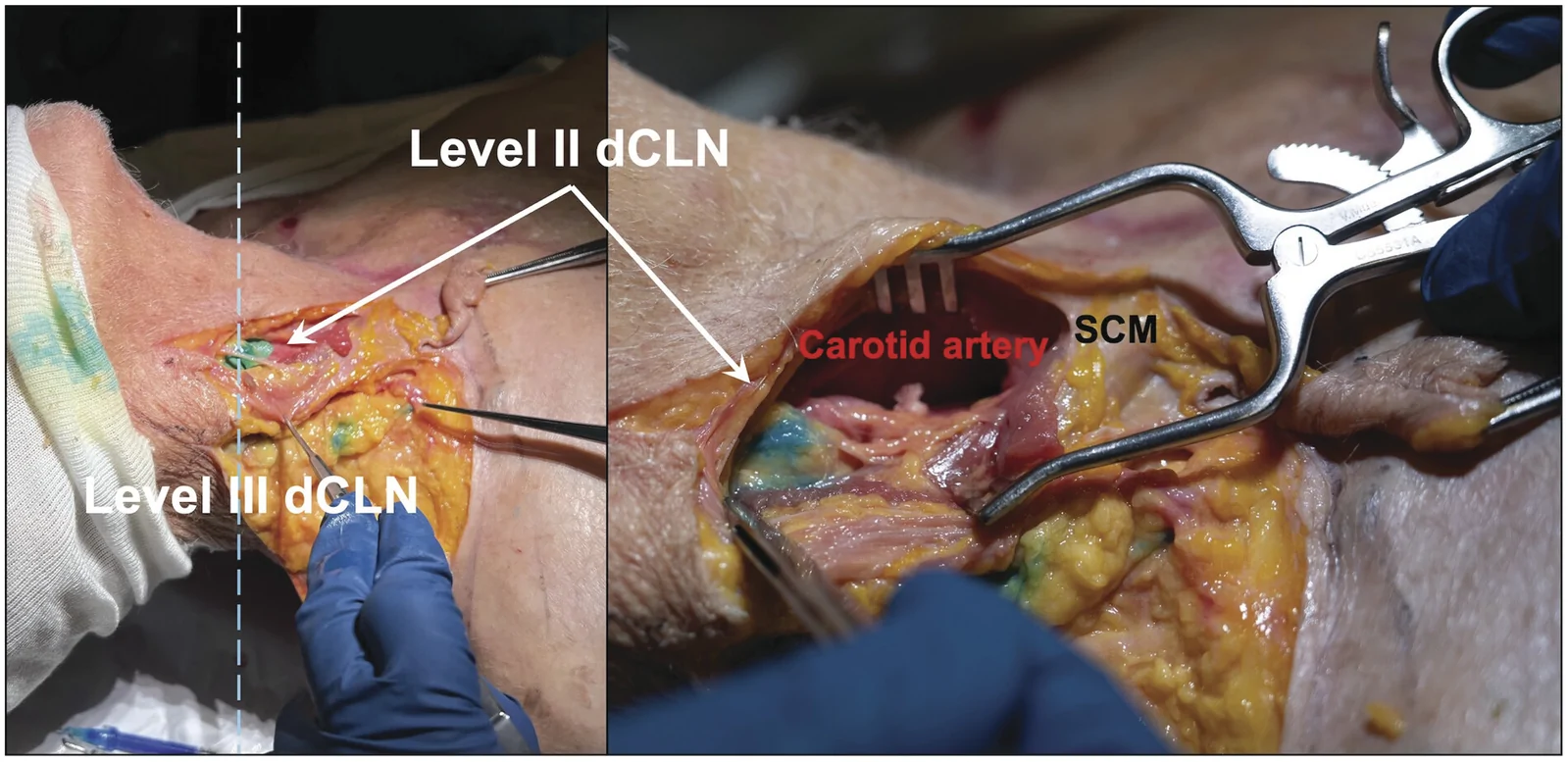
Plan for mapping the cerebral lymphatic posterior outflow pathway: The figure shows a possible route, divided into the retromandibular region (extending from the skull base to the digastric muscle); the deep digastric point, located just before the nodal levels; and the anatomical area of the deep cervical lymph node chain, extending from the digastric muscle to the clavicle. dCLN, deep cervical lymph node.

Cannulation was performed with a 33G needle using a mixture of acrylic paint, milk, and hydrogen peroxide. For more proximal dissection, the parotid gland, the mandibular branch, the zygomatic arch, and the deep musculature (pterygoids) were removed so that the internal jugular vein could be followed up to the base of the skull ( Fig. 2A–C ). A craniotomy window was created at the temporoparietal level, and 3 to 5 mL of ICG was injected as described above ( Fig. 3 ). Migration of fluorescent dye was monitored at the meningeal level, base of the skull, and at the cervical levels in the retromandibular region with a handheld imaging system (IC-Flow, Diagnostic Green Ltd). To confirm the presence of lymphatic tissues in these regions, samples of lymph nodes and lymphatic vessels were excised, preserved in 10% neutral buffered formalin, and processed for paraffin embedding. Sections were stained with hematoxylin and eosin (H&E) for immunohistochemical analysis using antibodies directed against CD31 and D2-40. Immunohistochemistry was also used to detect the presence of β-amyloid in CLNs, as well as lymph nodes of the groin, dissected from a cadaver with confirmed AD.

**Fig. 2:**
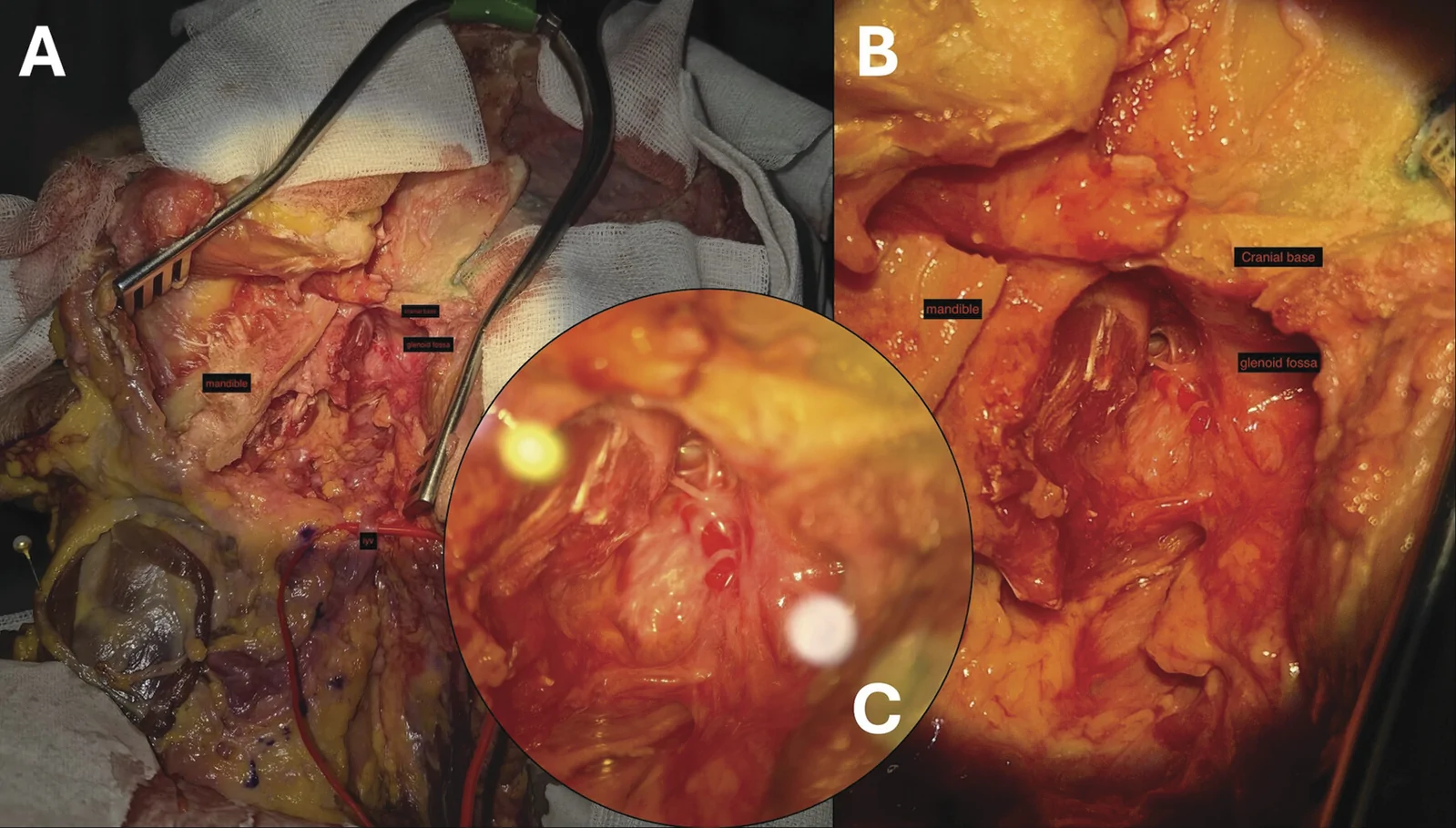
Mapping the retromandibular space from the skull base to the digastric muscle. ( **A** ) Retromandibular region exposed after removal of the parotid gland, zygomatic arch, mandible, and deep musculature. ( **B** ) Structures overlying the internal jugular vein, consistent with lymphatic structures, located immediately at the exit of the jugular foramen at the skull base. ( **C** ) Structures overlying the internal jugular vein, consistent with lymphatic structures.

**Fig. 3:**
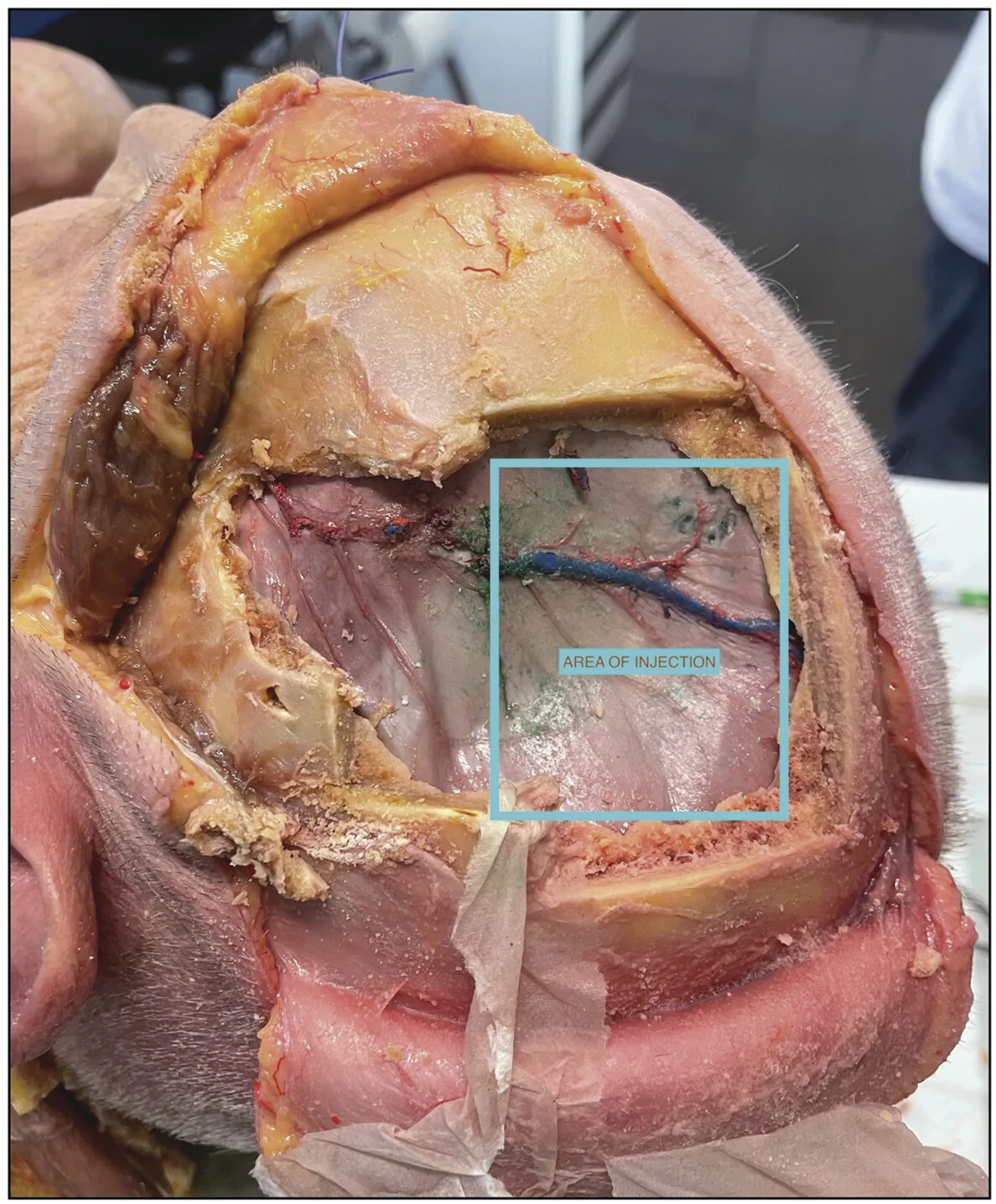
Site of indocyanine green injection at the dural level, in proximity to the middle meningeal artery.

### Simulated Lymph Node-to-Vein Anastomosis

The feasibility of LNVA was simulated by dissecting the right neck of a fresh cadaver (healthy donor) to the level of the hyoid cartilage, retracting the SCM, and splitting the carotid artery from the internal jugular vein to expose a Level II dCLN. A small capsular incision was created on the side of the node using a #11 blade. A previously dissected peripheral vein was then positioned adjacent to the lymph node without tension. Using robotic-assisted supermicrosurgery with the Symani Surgical System (Medical Microinstruments, Inc., Jacksonville, FL, United States). An end-to-side anastomosis between the vein and the capsular opening was performed using interrupted 10–0 nylon sutures.

## Results

**Video S2** ICG lymphography of dural lymphatic drainage into deep cervical lymph node. ICG, indocyanine green; LN, lymph node.


As expected, a reticular pattern was observed after ICG was injected into the dural venolymphatics. However, unlike in peripheral lymphatics, the ICG did not appear to be specific for lymphatic dural vessels, and instead there was dual staining of both veins and lymphatics. It was further observed that the infrared imaging was better able to demonstrate the deeper vascular structures compared with the photographic images.

Following the meningeal injection of ICG at the dura, fluorescence was observed progressing toward the skull base and was visible in the Level II dCLNs ( Video S2 [available in the online version only]). After injection of ICG into the parietal region, the lymphatic structures could be followed past the deep digastric point until they ultimately drained into the Level II CLNs ( Fig. 4 ). Candidate nodes at the deep digastric point and/or Level II were localized adjacent to candidate veins ( Figs. 5 6 7 ).

**Fig. 4:**
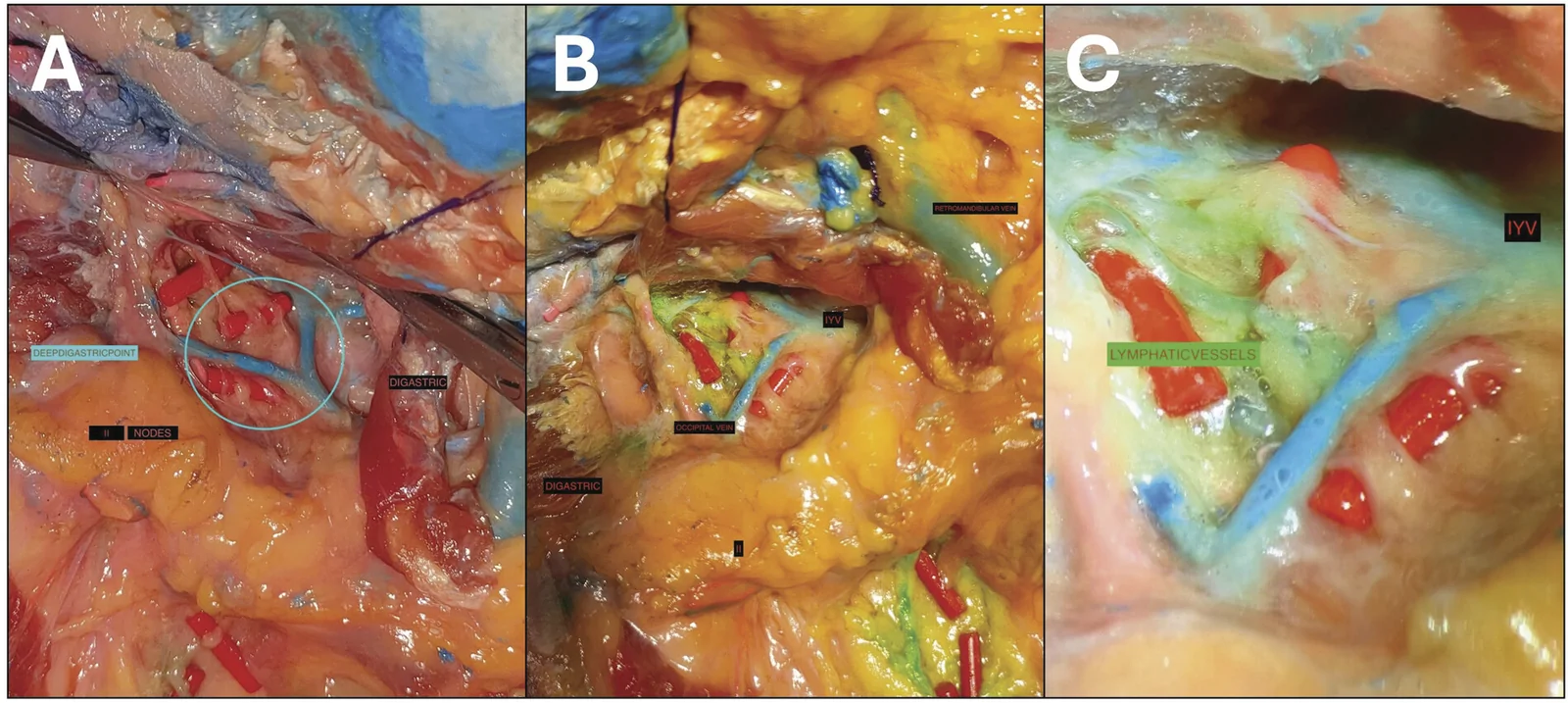
Visualization of the deep digastric point. ( **A** ) On retraction of the digastric muscle, a vein crossing posteriorly (occipital branch of the internal jugular vein) and lymphatic structures with a course originating from a more superior region were observed. ( **B** ) Lymphatic structures before Level II cervical lymph nodes (green), as well as more distal to them. ( **C** ) Microscopic view of lymphatic structures at the deep digastric point. IYV, internal jugular vein

**Fig. 5:**
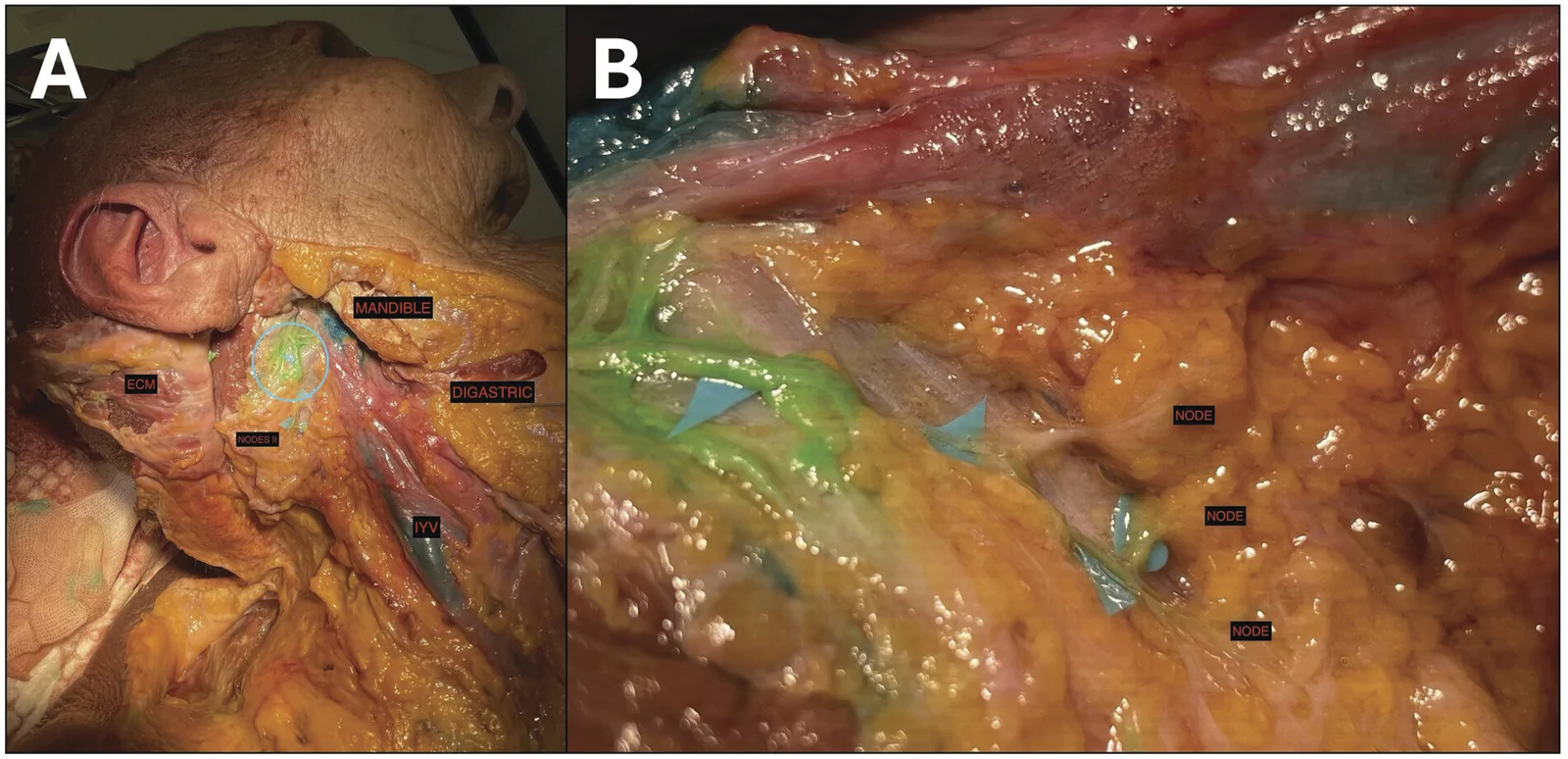
Visualization of lymphatics. ( **A** ) Mapping of lymphatic structures at Level II. ( **B** ) Detail showing how the lymphatic channels enter the cervical lymph nodes at Level II.

**Fig. 6:**
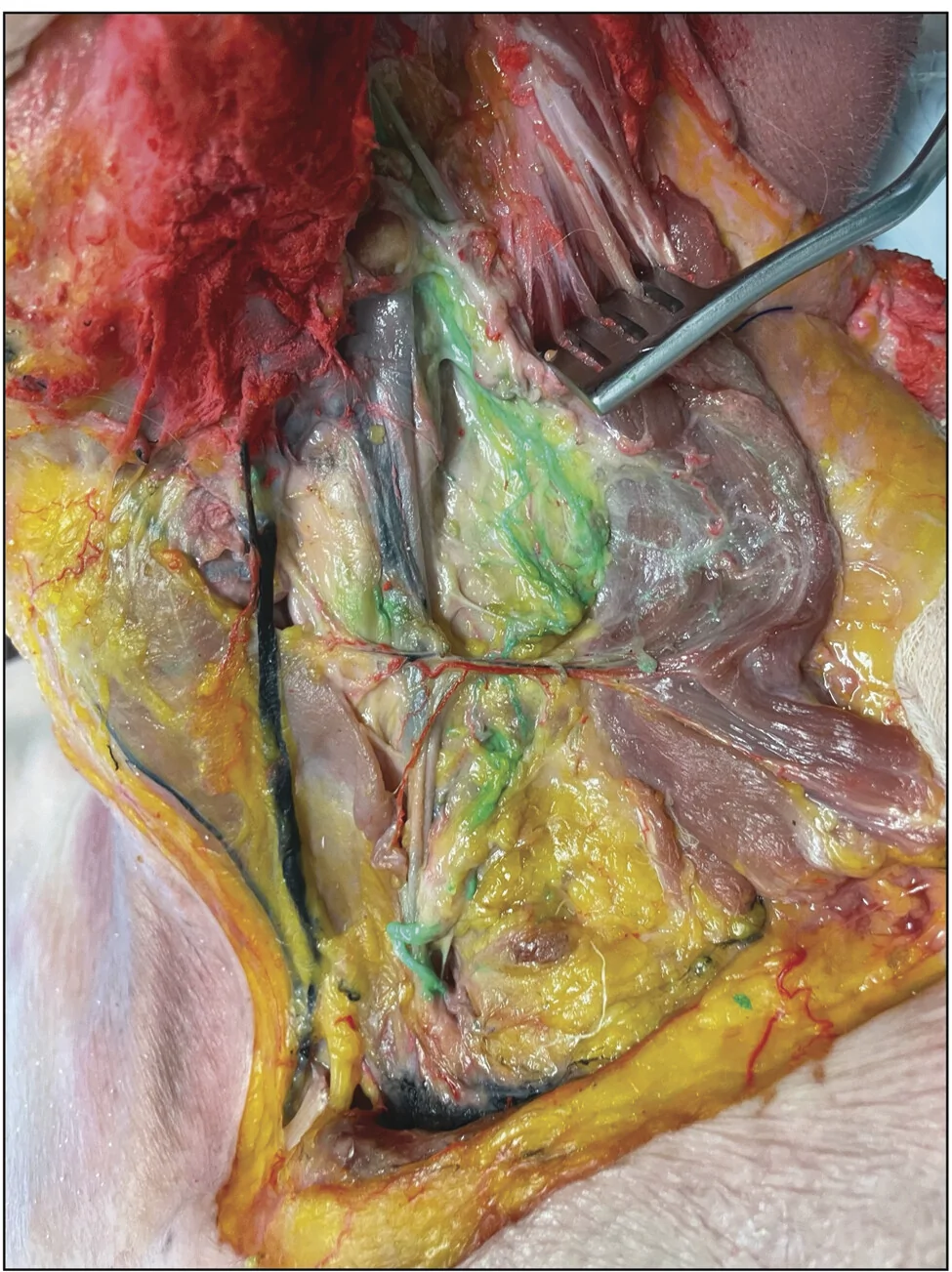
Lymphatic channels (green) in different levels.

**Fig. 7:**
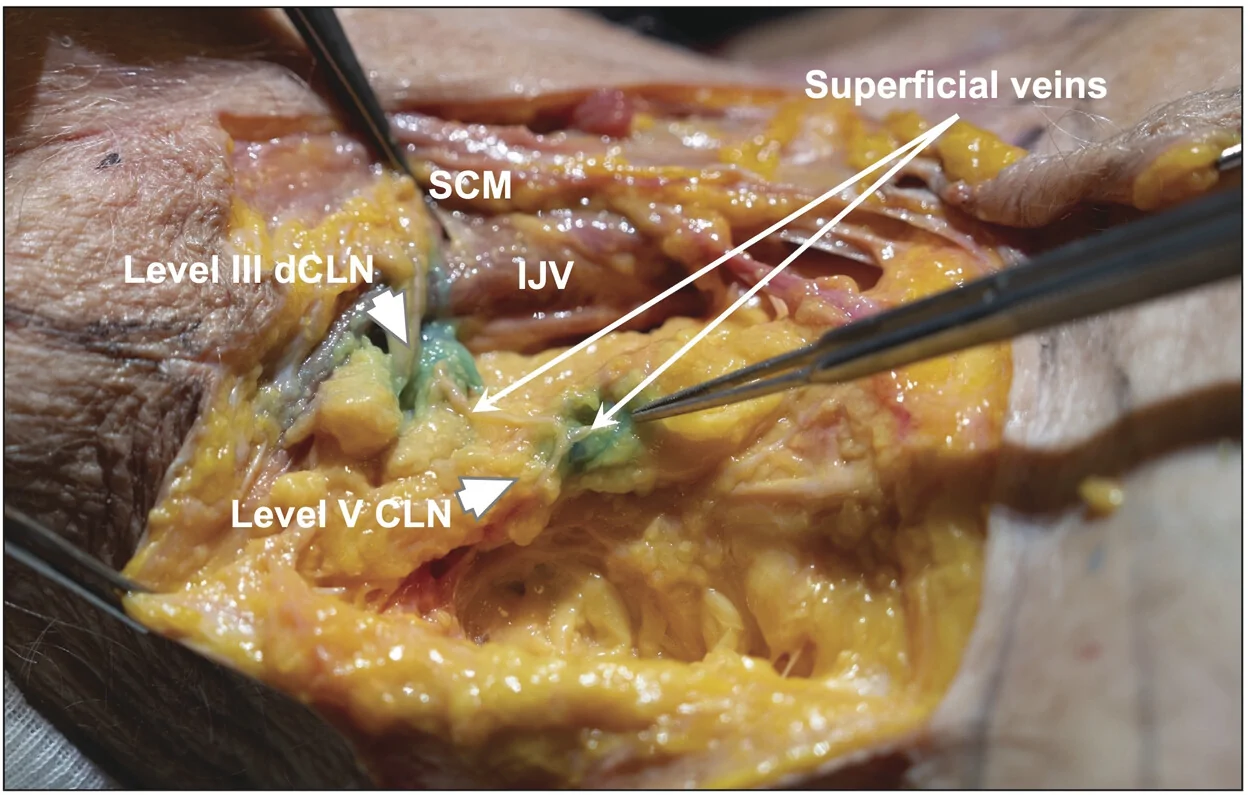
Identification of the Level III deep cervical lymph nodes (dCLNs), Level V CLNs, and superficial veins as labeled, in the right neck. The relative location of level III deep cervical lymph nodes lateral to the sternocleidomastoid muscle (SCM) and the internal jugular vein (IJV) is shown.

Staining of the Level II lymphatic tissue with H&E showed an autolytic node and lymphatic vessels ( Fig. 8A, B ). Immunohistochemistry with both anti-CD31/platelet endothelial cell adhesion molecule-1 (PECAM-1) and anti- D2-40/podoplanin antibodies confirmed the presence of lymphatic vessels ( Fig. 8C, D ).

**Fig. 8:**
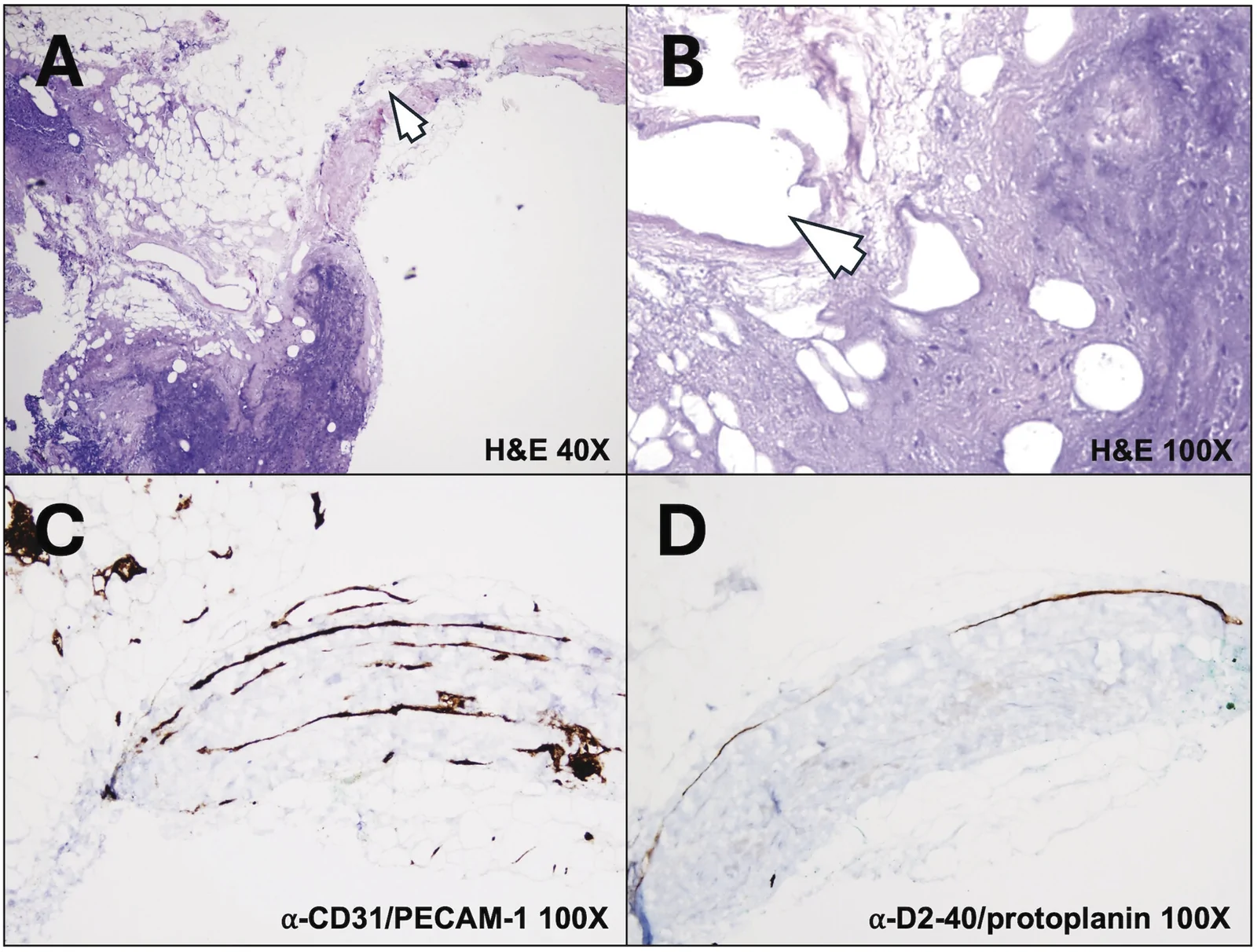
Histologic staining of Level II lymphatic tissue. ( **A, B** ) Hematoxylin and eosin (H&E) staining showing lymphatic vessels (arrows) and the autolytic node (40× and 100× magnification, respectively). ( **C** ) Immunohistochemistry with anti-CD31/PECAM-1 antibodies demonstrating the presence of vascular endothelium (100×). ( **D** ) Immunohistochemistry with anti-D2-40/podoplanin antibodies confirming the presence of lymphatic endothelium (100×).

Histologic analysis of lymph node specimens from the cadaver of a patient with a diagnosis of dementia showed that the dCLNs at Level II and a positive control stained positive for β-amyloid ( Fig. 9A–C ). The CLNs at levels III through V and those in the groin ( Fig. 9D ) were negative. In contrast, samples of the same regions from a cadaver without dementia were negative for β-amyloid.

**Fig. 9:**
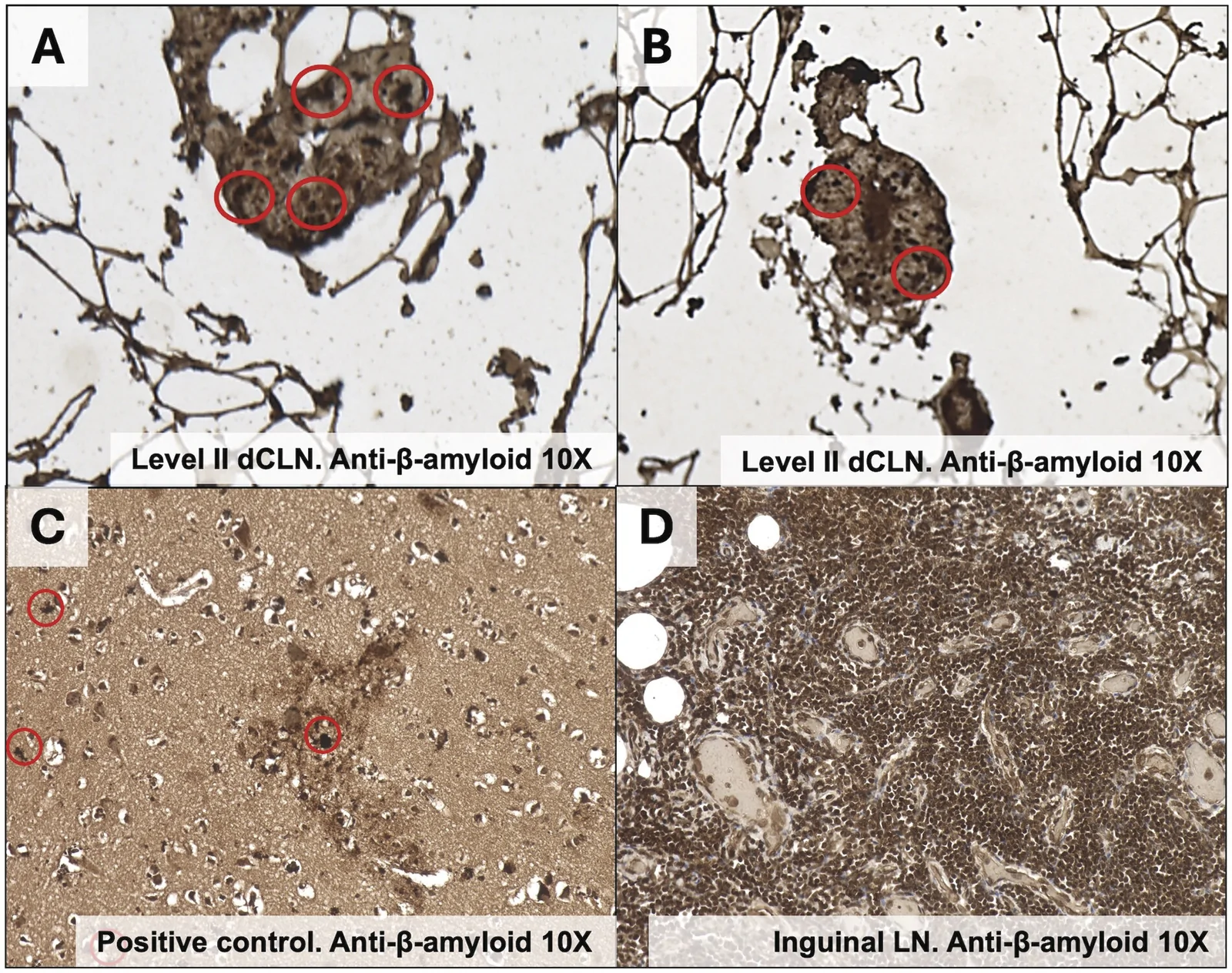
Immunohistochemical staining of cadaveric lymph nodes to detect β-amyloid protein. ( **A, B** ) Level II deep cervical lymph nodes (dLCNs). ( **C** ) A tissue sample known to express the β-amyloid antigen (positive control). ( **D** ) Inguinal lymph nodes. Red circles indicate aggregates staining positive for β-amyloid. All images are shown at 10× magnification.

The simulated LNVA was successfully performed, as planned, using robotic-assisted supermicrosurgery ( Figs. 10 and 11 ).

**Fig. 10:**
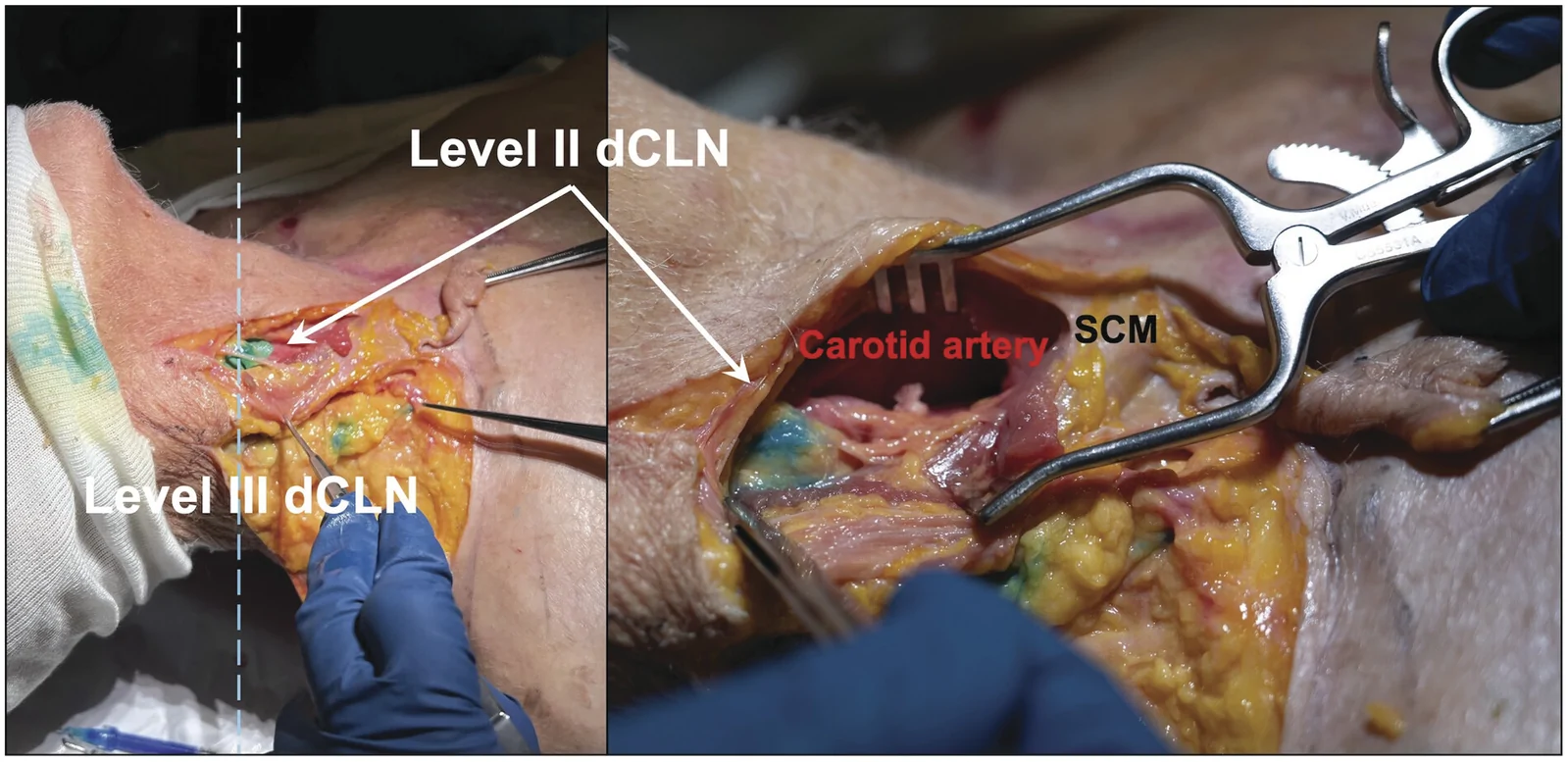
Preparation for simulated, robotic-assisted microsurgical lymph node-to-vein anastomosis. The level II deep cervical lymph nodes (dCLNs) are indicated by arrows. The carotid artery and sternocleidomastoid muscle (SCM) are also labeled to show their anatomic relationship to the Level II dCLNs.

**Fig. 11:**
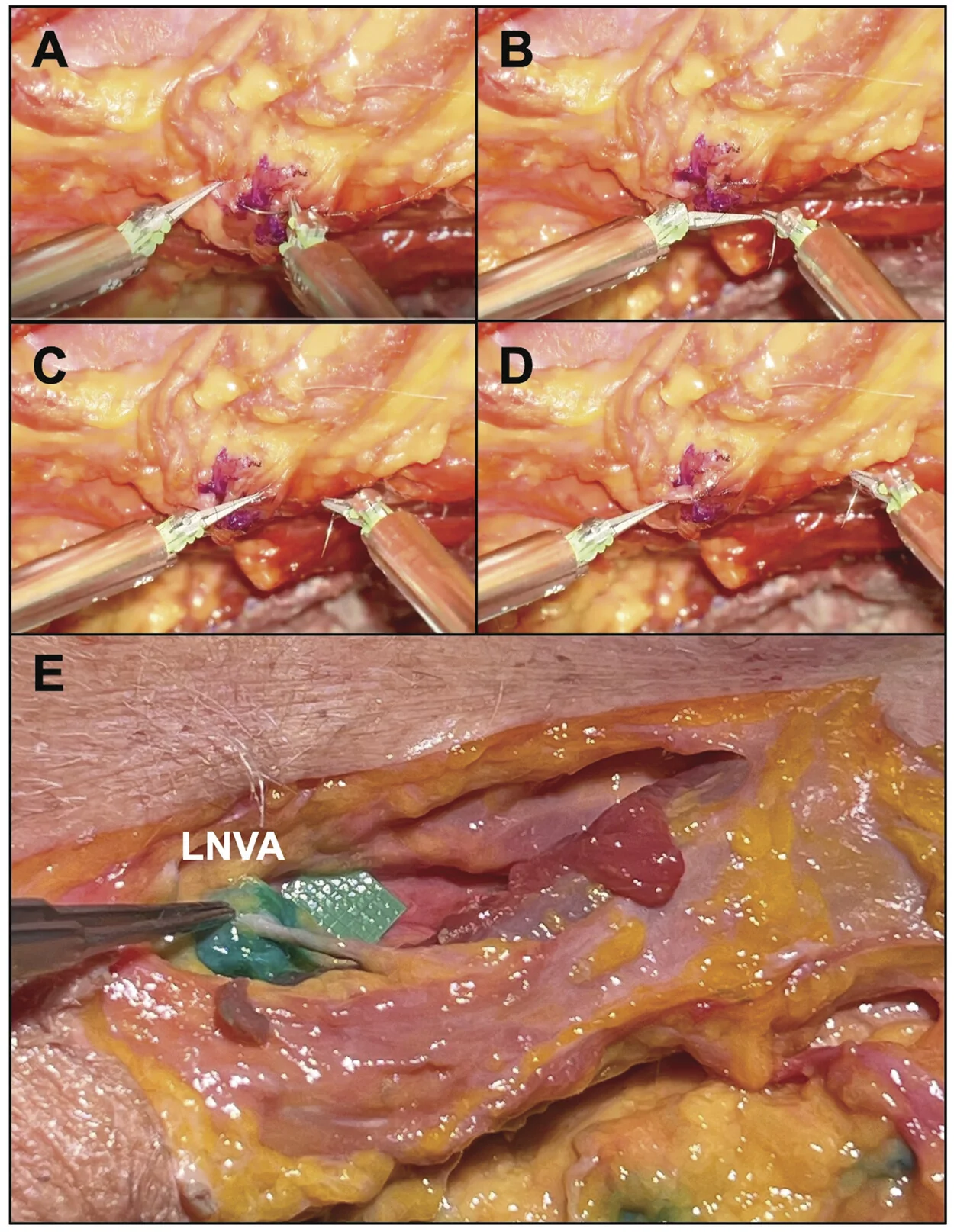
Simulated robotic-assisted microsurgical lymph node-to-vein anastomosis (LNVA) of the level II deep cervical lymph nodes. Panels ( **A–D** ) show the robotic, nanowristed instruments of the Symani being used to place and tie a suture. Panel ( **E** ) shows the completed LNVA.

## Discussion

In this cadaveric anatomical analysis, we initiated systematic mapping of the direct connections between the skull's dural lymphatics and the extracranial (cervical) lymphatic system in humans. ICG injection into the subcutaneous tissue of fresh cadaver specimens proved that the lymphatic vessels could take ICG into their lumen spontaneously, even postmortem. Gentle massage on the injection sites facilitated the transport of ICG inside the lymphatic vessels. 38 We speculate that the ICG injections into the dura demonstrated similar efficacy in depicting the lymphatics and in transporting ICG to the dCLNs.

Using this technique, we observed preliminary evidence of lymphatic drainage from the meningeal lymphatics at the skull base, at the level of the jugular foramen, to the Level II subdigastric CLNs. Histology-confirmed lymphatic structures—specifically Level II subdigastric nodes—were observed extending from the skull base through the retromandibular region toward the proximal CLNs, supporting the presence of a potential lymphatic drainage pathway from the cranial base to the neck.

One of the primary objectives of this study was to identify potential anatomical targets for the future development of surgical interventions for AD and other cognitive disorders hypothesized to arise from impaired clearance of neurotoxic macromolecules from the brain. One particularly promising candidate is the deep digastric point. This anatomical landmark is surgically accessible, located upstream of the CLNs, provides a direct anatomical route, and includes observed lymphatic structures and at least one vein potentially suitable for LVA.

In humans, the anatomy of the lymphatics distal to the digastric muscle is well-characterized, as the digastric muscle represents the superior boundary of cervical lymphadenectomy. If reduced cerebral drainage is indeed attributable to degeneration of lymphatic structures, intervention at this more proximal level may be justified. Such an approach would be particularly compelling if the presence of a direct lymphatic pathway from the jugular foramen through the deep digastric point to the origin of the dCLNs can be definitively established.

Based on our histological findings, the Level II CLNs may represent an additional surgical target. In lymph nodes excised from a cadaver with confirmed AD, we demonstrated β-amyloid accumulation in Level II CLNs, but not in nodes from Levels III to V or in the groin. This level is surgically practical, as the relevant target structures are consistently located superficially in the neck and can be accessed with relatively straightforward dissection beneath the SCM, within the space between the carotid artery and internal jugular vein. We further demonstrate the availability of suitable veins in close proximity to the Level II CLNs and successfully simulated an LNVA at this level using robotic-assisted microsurgery.

Direct anatomical mapping in cadaveric human heads builds on prior in vivo imaging studies in both humans and non-human primates. Multiple groups have used non-invasive magnetic resonance imaging (MRI) to confirm the presence of dural lymphatic vessels in these species, 24 26 39 40 41 42 and to delineate the human dural lymphatic network and its connections to the CLNs. 24 41 Our study contributes novel detail by helping to identify potential targets for surgical intervention.

Nevertheless, the development of surgical approaches to AD or other cognitive disorders must carefully weigh the relative advantages, limitations, and safety considerations of different cervical lymphatic levels ( Table 1 ). 6 16 29 These considerations underscore the need for continued basic and clinical research to define the safest and most effective targets before advancing LVA or LNVA procedures into clinical application.

**Table 1:** Potential considerations for selection of cervical lymph nodes for lymphatic procedures of the head and neck

Anatomic site	Advantages	Disadvantages
Retromandibular	• Most proximal	• Deep • Aggressive surgical approach • No small veins • Posterior route only
Deep digastric point	• Proximal • No superficial drainage • Oncological safety • Small veins available	• Deep • Posterior route only • No nodes available • LVA only
Jugulodigastric (II)	• Anterior and posterior routes • Proximal • Small veins available • LVA or LNVA	• Deep • Superficial drainage +++ • Oncological risk?
Middle jugular (III)	• Anterior and posterior routes • Many connections • LVA or LNVA	• Deep • Superficial drainage ++ • Oncological risk? • No small veins
Posterior triangle (V)	• Anterior and posterior routes • Easy approach • Most numerous nodes • Small veins available • LVA or LNVA	• Superficial drainage + • Oncological risk?
Submental (I)	• Easy approach • Small veins available • LVA or LNVA	• Superficial drainage • Oncological risk? • Anterior route only

Abbreviations: LNVA, lymph node-to-vein anastomosis; LVA, lymphovenous anastomosis.

We are aware of several groups that have made preliminary attempts at lymphatic surgical approaches to AD. 2 18 19 20 21 In parallel, rigorously designed ongoing and forthcoming clinical studies are expected to provide further insight into the feasibility of lymphatic surgery as a therapeutic strategy for AD. 43 In the interim, we reiterate our prior calls for careful, methodical investigation that appropriately considers surgical technique and its potential consequences. 2

Drawing on our experience with peripheral lymphatic procedures, we have previously hypothesized that LNVA or one-to-one, side-to-end LVA performed using supermicrosurgical techniques may be safer and more effective than gross dissection of the posterior bundle with unsystematic venous connections. 2 44 45 46 47 48 This cadaveric dissection validates the underlying anatomical rationale and demonstrates the feasibility of performing such bypass procedures at the dCLNs as a potential surgical approach for the treatment of AD.

### Study Limitations

This was an exploratory collaboration using a small number of specimens from seven healthy donors and one donor with confirmed AD. A larger cohort is required to rigorously identify and validate anatomical variations in the dural-to-cervical lymphatic pathways, which is critical for ensuring the safety and reproducibility of the proposed surgical interventions.

A major technical limitation was our finding that ICG staining was not specific to lymphatic vessels and may also stain venous structures in the dural lymphatics. Therefore, the findings are not conclusive and must be interpreted with care pending further study. Additionally, under the non-physiological conditions of these experiments, any observed fluorescence could reflect passive diffusion or injection artifact rather than true functional pathways. However, cadaveric models may still provide useful preliminary anatomical information or proof-of-concept data, especially when interpreted cautiously and supplemented with in vivo validation.

Methodologically, improvements in the cannulation technique and staining agents are needed. Mapping of the connections from the dural meninges to the skull base was preliminary and not exhaustive, and should therefore be viewed as a roadmap to inform future, formal investigation of potential recipient veins with a larger sample of cadavers. A detailed description of the surgical approaches and a thorough investigation of their safety are required before use in clinical practice.

### Conclusion

In summary, this study used ICG lymphography to confirm that human dural lymphatics connect to the dCLNs via the posterior route that drains through the jugular foramen. A number of potential candidates for surgical treatment of AD and other disorders potentially related to lymphatic obstruction in the head and neck were identified, and a robotic-assisted microsurgical LNVA was feasible. Further research is needed to build on these findings and develop safe and effective surgical approaches.
